# Life on a beach leads to phenotypic divergence despite gene flow for an island lizard

**DOI:** 10.1038/s42003-023-04494-x

**Published:** 2023-02-03

**Authors:** Richard P. Brown, Yuanting Jin, Jordan Thomas, Carlo Meloro

**Affiliations:** 1 School of Biological & Environmental Sciences, Liverpool John Moores University, Liverpool, L3 3AF UK; 2 College of Life Sciences, China Jiliang University, Hangzhou, 310018 People’s Republic of China

**Keywords:** Evolutionary ecology, Genetic variation

## Abstract

Limited spatial separation within small islands suggests that observed population divergence may occur due to habitat differences without interruption to gene flow but strong evidence of this is scarce. The wall lizard *Teira dugesii* lives in starkly contrasting shingle beach and inland habitats on the island of Madeira. We used a matched pairs sampling design to examine morphological and genomic divergence between four beach and adjacent (<1 km) inland areas. Beach populations are significantly darker than corresponding inland populations. Geometric morphometric analyses reveal divergence in head morphology: beach lizards have generally wider snouts. Genotyping-by-sequencing allows the rejection of the hypothesis that beach populations form a distinct lineage. Bayesian analyses provide strong support for models that incorporate gene flow, relative to those that do not, replicated at all pairs of matched sites. Madeiran lizards show morphological divergence between habitats in the face of gene flow, revealing how divergence may originate within small islands.

## Abstract (web summary)

Analyses of colouration, head shape, and genetic markers across pairs of neighbouring beach and inland habitats reveal replicated patterns of morphological divergence in the Madeiran wall lizard, despite gene flow between habitats.

## Introduction

Gene flow impedes divergence between populations by reducing differences in allele frequencies and facilitating the disruption of associations across loci. Nonetheless, detailed population genomic studies of some model organisms, including sticklebacks^1^ periwinkle snails^2^, and stick insects^3^, have shown how divergence and speciation can occur in the presence of gene flow. One scenario is the existence of strong divergent selection across different environments. In principle, divergence could accumulate around loci under selection while neutral loci will be homogenized by gene flow^4^. There is empirical evidence that neutral gene flow continues following colonization of a novel environment^5^. However, it is also possible that gene flow could be reduced at all loci mediated by, for example, selection against migration when this leads to lower fitness in the new environment^6–8^. The number of clear examples is relatively small and the identification of new model organisms is needed to obtain greater insights into the population genomics of divergence between environments.

Small oceanic islands have provided excellent models for studies of divergence and speciation, with lizards being one of the more frequently-studied vertebrates. Many island species occupy a greater variety of environments than continental counterparts and this can provide opportunities for examining divergence and gene flow between habitats. To date, there seem to be few examples that demonstrate within-island adaptive divergence with ongoing gene flow. Instead, many studies have shown that either historical or current interruptions to gene flow have contributed to population divergence and speciation^9–12^, with volcanic events such as debris avalanches and major lava flows often being implicated^11,13,14^.

A better understanding of how within-island divergence originates may also be important in explaining how island communities develop. Adaptive responses to different microhabitats seem to partially explain the existence of sets of species comprising distinct ecomorphs within some islands^15,16^ although, as described above, interruption of gene flow by spatial isolation is also likely to have been important^17^. In addition to these historical approaches, population-level studies of species showing incipient divergence within a single island could provide better insights into whether population isolation is a prerequisite for within-island evolution. These studies are facilitated by methods that can examine historical and current gene-flow based on the coalescent^18–20^, with recent approaches being highly suitable for use with genomic data^21^. Here we examine habitat-associated divergence between populations and the degree of gene flow between them.

The first aim was to test for morphological and colour divergence in a lizard between several pairs of similar adjacent habitats: parallel patterns of divergence at different locations can substantiate the hypothesis of divergent selection^22^. Divergence in dorsal colour was studied as this has been found to vary in a way that appears to enhance crypsis on different backgrounds in other small vertebrates, such as lizards and mice^23–25^. Morphological divergence is less well-known over such short distances but has been detected for this species^26^. We found consistent patterns of divergence in both of these groups of characters, replicated across sample locations, which provided a platform for testing our main hypothesis that this had occurred in the face of gene flow. We tested genomic divergence between matched pairs of adjacent habitats and detected ongoing gene flow in every case.

## Results

### Colour variation

Beach (B) individuals had lower luminance (i.e., were darker) on average than inland (I) individuals. A two-way MANOVA on log_10_-transformed R, G and B luminances of the 6 colour characters indicated that habitat, locality and habitat-locality interaction were all highly significant (habitat, Pillai’s Trace = 0.450, F_6,201_ = 27.44, *P* < 0.001; locality, Pillai’s trace = 0.479, *F*_18,609_ = 6.43, *P* < 0.001; interaction, Pillai’s trace =0.285, F_18,609_ = 3.55, *P* < 0.001). The effect size for males was considerably greater for habitat (partial η^2^ = 0.45) than for locality (partial η^2^ = 0.16) and habitat-locality interaction (partial η^2^ = 0.10). For females, habitat, locality and their interaction were also highly significant (habitat, Pillai’s trace = 0.463, *F*_6,105_ = 15.08, *P* < 0.001; locality, Pillai’s trace = 0.399, *F*_18,321_ = 2.74, *P* < 0.001; interaction, Pillai’s trace = 0.338, *F*_18,321_ = 2.26, *P* = 0.003). Again, the effect size was much greater for habitat (partial η^2^ = 0.46) than for locality (partial η^2^ = 0.13) and habitat-locality interaction (partial η^2^ = 0.11).

**Fig. 1:**
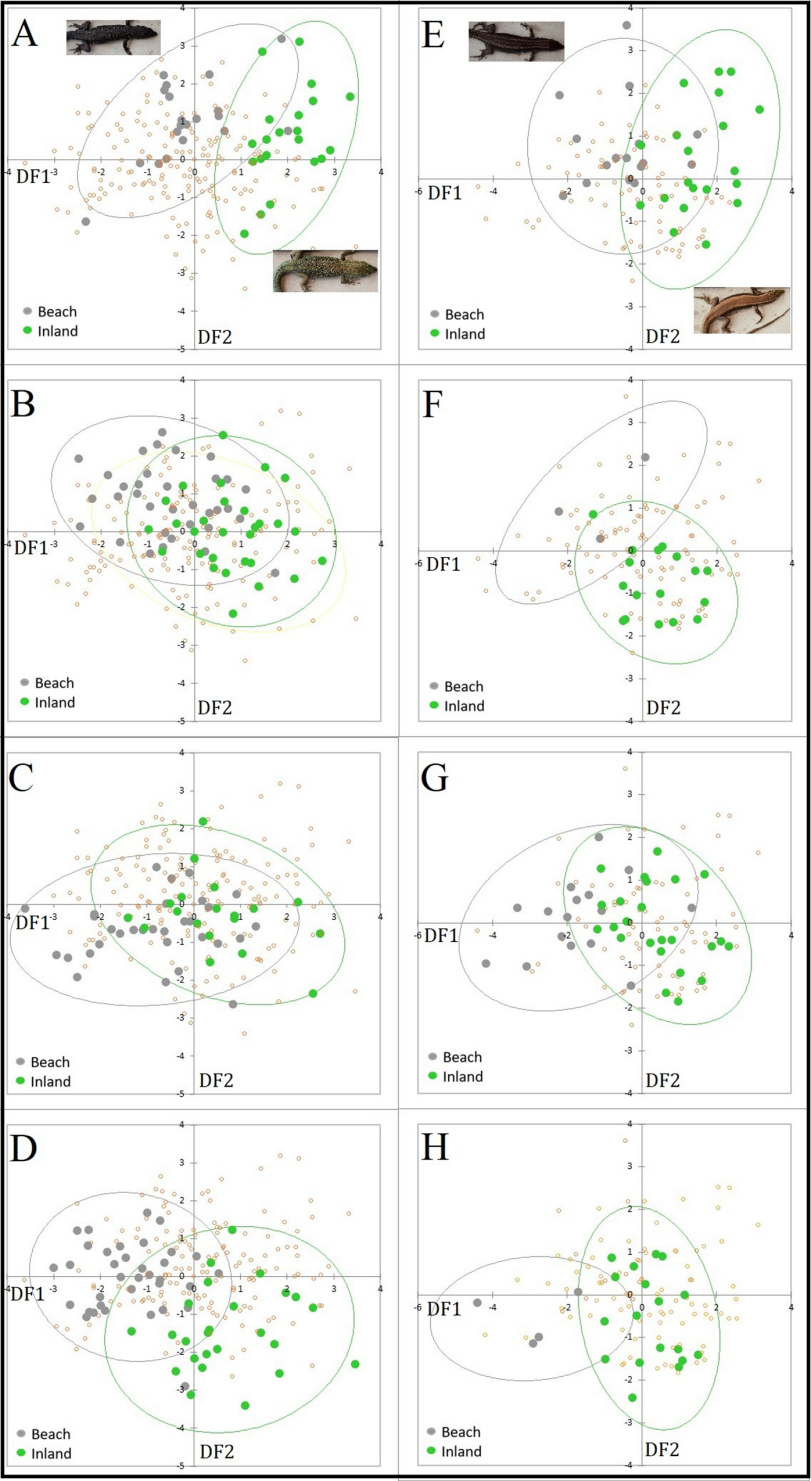
Lizard luminance. Plots of scores from the male (**A**–**D**) and female (**E**–**H**) discriminant function analyses (DFA) of luminance, with 95% confidence ellipses. For males, DF1 and DF2 represented 55.7 and 28.6% of the variation; corresponding values for females were 65.8 and 15.4%, respectively. While the DFAs were carried out across all eight sites, just the scores for matched pairs of beach and inland sites are emphasized in each separate plot for clarity: plots **A** and **E** correspond to locality 1, plots **B** and **F** to locality 2, **C** and **E** to locality 3, and **D** and **H** to locality 4. Additional, transparent points on each plot show scores for individuals from other sites. Inset photographs in the uppermost Fig. **A**, **E** show representative inland and beach individuals, which were similar at all localities. Sample sizes are given in Supplementary Table 1.

DFAs revealed that most of the variation across the eight locality/habitat groups was expressed by the first two discriminant functions (DFs). Concordant with the finding of a significant habitat effect (above), the individual DFA plots (representing 84.3% of the variation in males and 80.2% in females) showed that beach individuals were clearly divergent on male and female DF1 axes with largely negative scores at all localities (Fig. 1). The luminance characters 1–5 had positive variable coefficients for DF1 for males (character 6 was close to zero; see Supplementary Table 2), showing that lower luminance was associated with B relative to I individuals (Fig. 1A–D). The directions of these B/I differences were consistent across all four localities. Characters 1, 4 and 5 showed high positive variable coefficients for females, while the remaining characters had lower negative coefficients, closer to zero. The DFA scores (Fig. 1E, F) suggested that females were also generally darker at the beach sites (lower luminance).

### Morphology

Two-way ANOVA on head size in females revealed effects of habitat (*F*_1,108_ = 4.30; *P* = 0.041; partial η^2^ = 0.038), locality (*F*_3,108_ = 3.66, *P* = 0.015; partial η^2^ = 0.092), and locality–habitat interaction (*F*_3,108_ = 2.78, *P* = 0.044; partial η^2^ = 0.072). Analysis of male head size revealed greater within-group variation (due to larger size differences in samples) and so five small outlying males were removed prior to the two-way ANOVA to meet the assumptions of normality and homoscedasticity. The ANOVA revealed a habitat effect that was close to the 5% significance level (*F*_1,200_ = 3.70, *P* = 0.056; partial η^2^ = 0.018) a significant difference between locations (*F*_1,200_ = 3.92, *P* = 0.010; partial η^2^ = 0.056) and a large interaction effect (*F*_1,200_ = 10.09, *P* < 0.001; partial η^2^ = 0.131). Although variation was significant, there were no consistent patterns of variation in mean size between habitats or between localities. Inland males from locality 2 (Supplementary Fig. 5A) were the largest individuals, on average, while among females, specimens from the B site at locality 1 were the smallest (see Supplementary Fig. 5B).

For male head shape, two-way MANOVA of the first 23 PCs revealed that habitat had the largest effect size and was highly significant (Pillai’s Trace = 0.477, *F*_23,183_ = 7.26, *P* < 0.001; partial η^2^ = 0.477), while locality (Pillai’s trace = 0.713, *F*_69,555_ = 2.51, *P* = 0.238; partial η^2^ = 0.238) and interaction (Pillai’s trace = 0.547, *F*_69,555_ = 1.79, *P* = 0.182; partial η^2^ = 0.182) were not significant. For the first 21 PCs analyzed for females, habitat again had the largest effect size and was highly significant (Pillai’s Trace=0.488, *F*_21,88_ = 3.99, *P* < 0.001; partial η^2^ = 0.488), locality was significant (Pillai’s trace = 0.856, *F*_63,270_ = 1.71, *P* = 0.002; partial η^2^ = 0.285), but the habitat-locality interaction was not significant (Pillai’s trace = 0.693, F_63,270_ = 1.29, *P* = 0.089; partial η^2^ = 0.231). (Note that, as for dorsal luminance, Pillai’s trace test statistic was used because two male and one female input PCs appeared to deviate from normality, with inequality of covariance matrices also being detected).

**Fig. 2:**
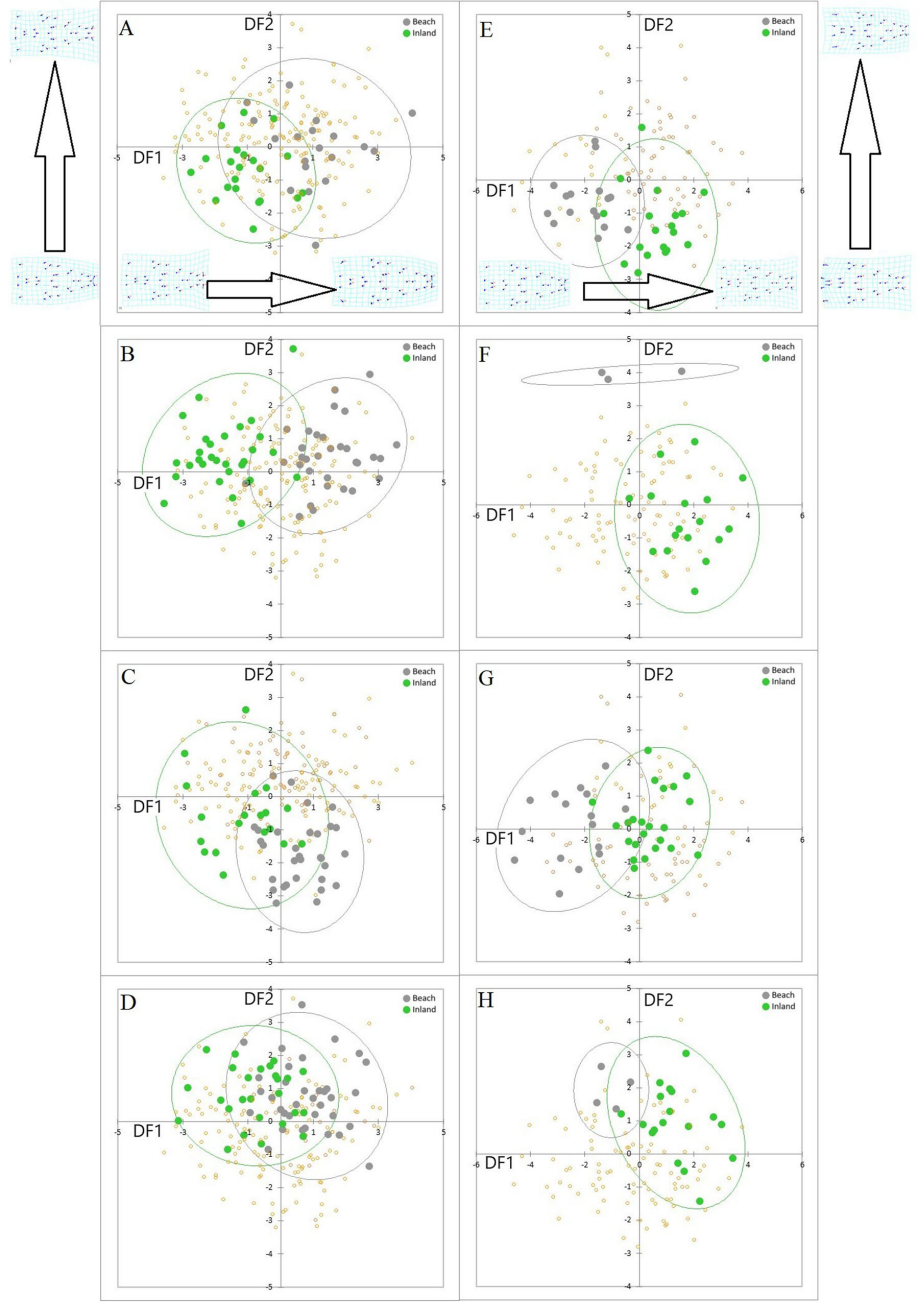
Lizard head shape. Plots of DF1 and DF2 scores from discriminant function analyses of head shape for localities 1-4 for the male (**A**–**D**) and female (**E**–**H**), with 95% confidence ellipses. Plots **A** and **E** correspond to locality 1, plots **B** and **F** to locality 2, **C** and **E** to locality 3, and **D** and **H** to locality 4. For males, DF1 and DF2 represented 38.5 and 18.4% of the total variation and positive DF1 scores correspond to wider snouts while corresponding values for females (plots **E**–**H**) were 26.2 and 20.3%, respectively, with negative DF1 scores corresponding to wider snouts (the deformation grids indicate how head shape changes along the two axes). The DFAs were carried out across all eight sites, but just the scores for matched pairs of beach and inland sites are highlighted in each plot for clarity (the smaller, transparent points show remaining scores for individuals from other sites). Sample sizes are given in Supplementary Table 1.

The first two DFs from the DFA (representing 56.9% of total variance) illustrated how females were divergent between B and I sites at all localities and the direction of the divergence was the same in all cases, i.e., individuals at B localities had a broader snout than I individuals (Fig. 2). A parallel pattern was found for males, where the first two DFs represented 56.5% of the total variance (56.5%) and males had broader snouts at B sites (Fig. 2). Hence the pattern of divergence is replicated across sexes and across four localities. The only slight deviation was for females at locality 2 (Fig. 2F). Beach/inland divergence was still present, but mainly on DF2 rather DF1, although it should be noted that the female beach sample size was extremely small in this case.

### Habitat variation

As expected, B and I sites differed both in terms of the substrate RGB scores (i.e., lower luminance at grey shingle beach sites) and percentage vegetation cover (typically 60% cover at land sites, zero cover at beach sites). For luminance, a two-way MANOVA on log_10_ RGB values indicated significant effects for all terms in the model (habitat, Pillai’s Trace = 0.871, *F*_3,69_ = 154.70, *P* < 0.001; locality, Pillai’s trace = 0.270, *F*_9,213_ = 2.34, *P* < 0.001; interaction, Pillai’s trace = 0.442, *F*_9,213_ = 4.09, *P* < 0.001). Habitat showed a very large effect size (partial η^2^ = 0.87) relative to locality (partial η^2^ = 0.09) and the interaction (partial η^2^ = 0.15). (As before, Pillai’s trace test was used due to evidence of inequality of covariance matrices, although all residuals were normally distributed).

**Fig. 3:**
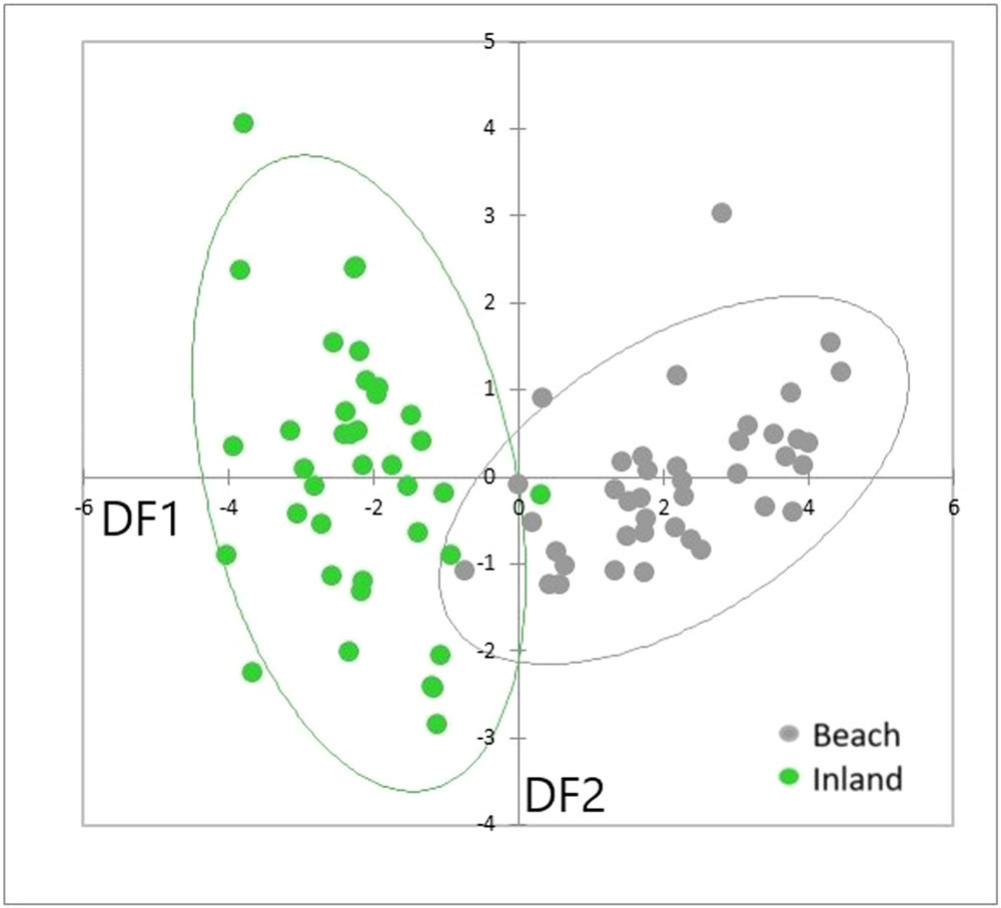
Substrate luminance. Scatterplot of scores from the first two discriminant functions, DF1 (89.2% of total variation) and DF2 (8.9% of total variation) of RGB values recorded from quadrat sampling of the substrate at beach and inland sites. The data were grouped by the eight sample sites in the analysis, but the points are labelled as either beach and inland sites for simplicity. The corresponding 95% confidence ellipses were obtained for the two habitat types (pooled across localities). Sample sizes are given in Supplementary Table 1.

The DFA on the three RGB values showed separation of B and I sites, irrespective of locality (Fig. 3) with minimal overlap indicating clear differences in substrate luminance. The variable coefficients were strongly positive for blue and strongly negative for red (green was intermediate) for DF1 (Supplementary Table 3) and show that blue values were much greater and red values much lower for sites with shingle beach substrates, relative to the inland sites.

No vegetation was found in any of the B samples, while median vegetation cover was greater than 60% at all I samples (see Supplementary Fig. 4).

### GBS analysis

After filtering, a total of 19311 SNPs were identified in 4135 tags from 93 individuals from the eight locality-habitat groups and corresponded to the ALLSNP data. A thinned dataset (4131 SNPs) was obtained by removal of SNPs that showed patterns expected under selection (see pcadapt analysis below), together with sampling of one SNP per tag.

#### Evidence of selection

A total of 52 outlier SNPs were detected within the ALLSNPS data, after Bonferroni correction, using pcadapt. Of these outliers, only four SNPs showed a significant association with habitat type, but none of these were located on the same tag. Three of these were significant for 7/10 or fewer bayenv replicates. One SNP showed a significant association with habitat type for 9/10 replicates, although none of the other SNPs on the same tag were outliers.

#### Spatial structuring

**Fig. 4:**
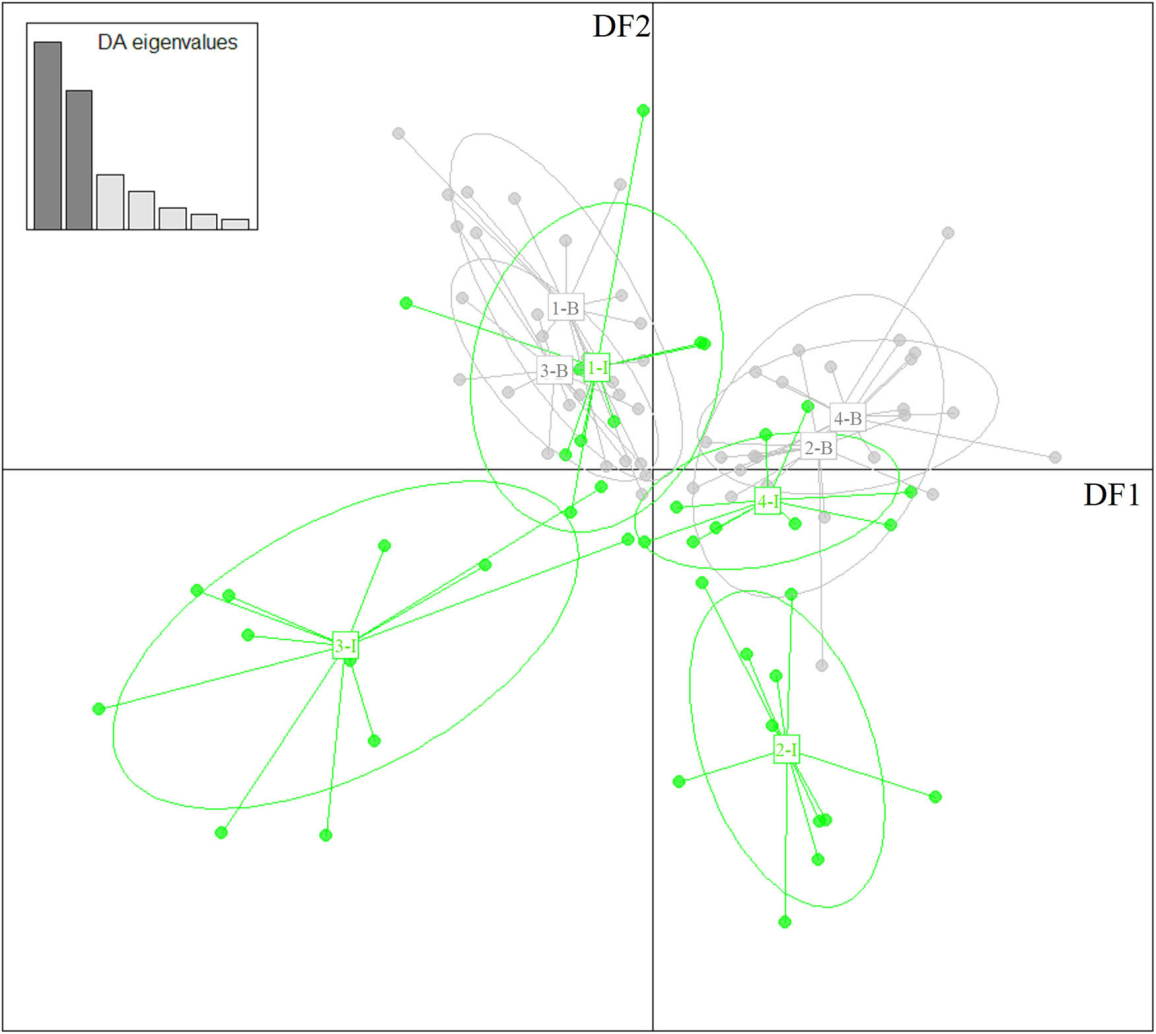
Genomic variation between sites. Scatter plot of scores from Discriminant Analysis of Principal Components analysis of the genomic data. Population labels indicate the locality and beach (B) or inland (I). The first discriminant function, DF1, represents 40.1% of the variation, while DF2 represents 29.9%. The inset plot (DA eigenvalues) shows the relative decline in variance explained by successive Discriminant functions from 1 to 7. Sample sizes are given in Supplementary Table 1.

Pairwise F_ST_ summary statistics are provided in Supplementary Table 4. The DAPC analysis of the ALLSNP dataset provided some evidence of divergence between localities and between habitats but there was no consistent pattern of B/I divergence. Eighteen PCs were favoured as input for the DFA following cross-validation (MSAR = 56.63%, RMSE = 0.457). The first two discriminant functions (DF1 and DF2) captured most of the variation (70.0%; Fig. 4). There was some regional separation of groups along DF1: the two south coast localities (1 and 3) appeared divergent from the two north/east coast sites 2 and 4. On DF2, the 2-I and 3-I individuals could be clearly discriminated from corresponding 2-B and 3-B lizards from the same localities, and from B/I lizards from the other locality on the same coast.

The sPCA on the thinned dataset revealed significant local structuring among neighbours within the localities (observation= 34.39, *P* = 0.0002), as well as significant global structuring (observation= 29.02, *P* = 0.0010).

#### Relationships among localities/habitats

Treemix provided no support for the hypothesis of two main lineages, comprising respective B and I populations (Supplementary Fig. 5). South coast sites 1-B and 3-B grouped together suggesting a possible relationship, but bootstrap support was very weak. Overall, the analysis grouped sites 1-I, 1-B, and 3-B relative to the remaining sites but weak bootstrap support and a lack of any clear geographical pattern, suggested little or no phylogeographic structure.

#### Historical gene flow

AIC values most strongly supported the scenario of divergence followed by two different periods of gene flow at each of the localities, based on the INDSNPs datasets (exactly the same pattern was detected for the all ALLSNP datasets) (Table 1). The model of divergence with no gene flow provided the worst fit to the observed SFS at all localities.

**Table 1:** Differences between observed and simulated site frequency spectra for three gene flow models.

	Locality			
Gene flow model	1	2	3	4
TWOGFLOW	9.35 (0)	4.84 (0)	6.60 (0)	6.49 (0)
ONEGFLOW	111.57 (460.7)	108.44 (692.0)	108.44 (463)	86.27 (361.4)
NOGFLOW	202.07 (871.4)	272.66 (1221.4)	179.91 (786.2)	154.67 (670.4)

Fastsimcoal2 comparisons between models for independent SNP datasets for each locality. Differences in likelihood between observed and simulated site (allele) frequency spectra are given for all models and localities. Values in parentheses provide ΔAIC units between each model and the best model (i.e., TWOGFLOW at all localities). Probabilities with respect to no difference in minimization of information loss are not provided because even for the smallest ΔAIC between models (i.e., 361.4 for ONEGFLOW at locality 4) the probability for ONEGFLOW relative to TWOGFLOW is extremely small (1.1 × 10^−157^). Sample sizes for localities 1–4 were: 17, 17, 20, and 17, respectively.

The favoured model (TWOGFLOW) indicated lower migration rates per individual immediately following divergence, followed by a more recent period of higher migration rates. This pattern was replicated at all four localities (see Table 2 for details). The mean estimated timing of the initial split between beach and inland populations) is quite variable, ranging from 175012 generations at locality 1 to 1709064 generations at locality 4 (although 95% bootstrap intervals overlap for these localities). For context, while we could find no published studies of generation time in *Teira dugesii*, it has been estimated at 2.1 years in another wall lizard^27^. Mean migration rate estimates were mostly higher from inland to beach than vice-versa.

**Table 2:** Estimated divergence times and migration rates between beach/inland habitats at the for localities.

	Ancient period			Recent period		
		Migration rates			Migration rates	
Locality	Time	Beach->Inland	Inland->Beach	Time	Beach->Inland	Inland->Beach
1	175012 (27791, 641032)	<1 × 10^−6^ (<1 × 10^−6^, 8.37 × 10^−4^)	3.58 × 10^−5^ (9.12 × 10^−6^, 5.11 × 10^−5^)	1046 (1245, 2069)	9.81 × 10^−4^ (<1 × 10^−6^, 3.13 × 10^−3^)	1.07 × 10^−3^ (4.26 × 10^−4^, 4.52 × 10^−3^)
2	246721 (43310, 738041)	<1 × 10^−6^ (<1 × 10^−6^, 5.99 × 10^−4^)	2.55 × 10^−5^ (<1 × 10^−6^, 2.29 × 10^−3^)	1915 (192, 3502)	6.47 × 10^−4^ (<1 × 10^−6^, 2.29 × 10^−3^)	7.26 × 10^−4^ (3.48 × 10^−4^, 3.36 × 10^−3^)
3	797338 (104552, 854471)	<1 × 10^−6^ (<1 × 10^−6^, <1 × 10^−6^)	<1 × 10^−6^ (<1 × 10^−6^, <1 × 10^−6^)	537 (320, 595)	2.34 × 10^−4^ (2.28 × 10^−4^, 5.37 × 10^−4^)	1.64 × 10^−2^ (1.10 ×10^−2^, 3.23 ×10^−2^)
4	1709064 (17383, 21810456)	<1 × 10^−6^ (<1 × 10^−6^, 8.32 × 10^−4^)	5.32 × 10^−5^ (<1 × 10^−6^, 1.08 × 10^−4^)	1095 (107, 1797)	9.08 × 10^−4^ (4.80 × 10^−4^, 6.40 × 10^−3^)	1.01 × 10^−3^ (<1 × 10^−6^, 3.92 × 10^−3^)

Fastsimcoal2 parameter estimates and corresponding bootstrap values for the model allowing two periods of gene flow for the ALLSNP dataset. Under the favoured model (see Table 1), early divergence was proceeded by lower beach-inland migration rates (i.e., during the “Ancient period”). This was followed by a “Recent period” of relatively higher migration rates. Each of these periods was estimated to have begun at a specific Time (estimates in generations before present) while Beach->Inland and Inland->Beach migration rates represent the estimated probabilities of a lineage within one of these habitats moving to the other habitat, per generation, going forwards in time. The 95% bootstrap confidence intervals for all estimates are given in parentheses. Sample sizes for localities 1–4 were: 17, 17, 20, and 17, respectively.

## Discussion

This study demonstrates morphological divergence between lizards found on distinct grey shingle and boulder beaches and those from inland environments less than 1 km away. Most notably, this pattern is replicated at four unconnected beaches across Madeira. The direction of change between habitats for both head morphology and dorsal colour is repeated at all localities and for both males and females, i.e., generally broader snout and darker dorsal coloration at beach sites, providing support for the hypothesis that divergent selection between the two environments is sufficient to overcome gene flow. Ongoing gene flow between environments was detected at all localities and showed similar patterns, including greater gene flow now than in the past and higher gene flow from inland to beach than the other way around. The genomic data did not support the hypothesis that beach-inland divergence was due to distinct evolutionary lineages occupying the different environments.

Beach/inland morphological divergence had been reported previously at one of our localities (Caniço^26^) but our findings differ in detail. The previous study described differences in perceived darkness of the lizards and relative digit and tail length, but not in relative head width (after adjustment for body length). Hence, the variation that we found in head shape was largely unexpected. Although the morphological divergence in colour and head morphology is highly statistically significant, it is also clear that there is considerable morphological overlap between habitats for both of these sets of traits. This would largely be expected under gene flow.

Genomic analyses of models of divergence showed similarities at all matched pairs of beach and inland sites. The same gene flow scenario was supported in each case: initial divergence following the assumed colonization of the beach from the inland. Beach-living is fairly unusual in this lizard group so it is assumed that *T. dugesii* invaded inland habitats, similar to those occupied by its ancestor, immediately after island colonization (see ref. ^28^). This was proceeded by a long period of lower gene flow, prior to higher gene flow during more recent times. High levels of allelic exchange between habitats seem unquestionable due to the extremely abundant and ubiquitous nature of this species, particularly in coastal areas. The only likely unsuitable habitat between our beach and inland sites were one or more narrow roads but these should not present a barrier, as we frequently saw lizards on/around roads.

The asymmetric gene flow with generally higher rates from inland to beach sites would be predicted between a large metapopulation (the main island) and a peripheral habitat (i.e., the beach). In contrast, the finding that historical gene flow is relatively lower than more recent gene flow is less easy to explain. Recent gene flow estimates were generally one or more orders of magnitude higher than equivalent estimates during the ancient period of gene flow and are again substantiated by being repeated across the four study areas. The expectation under ongoing divergence might be higher gene flow to begin with, as the beach habitat is colonized, followed by a decrease over time due to evolution of assortive mating at the ecotone^29^ and/or reduced migration between beach and inland habitats. There is no obvious historical scenario that might explain this, although it would fit an island-mainland model, involving the creation of relatively isolated coastal demes by colonization from inland habitats. Migration rates could have subsequently increased after changes in coastal topography/sea-level. Sea-level fluctuations have had an impact on coastal communities^30^ with dramatic changes in habitat availability expected even during the recent 18,000–6000 years BP period, when ~130 m rises in sea level were evident^31^.

The hypothesis of divergent selection obviously requires that variation in dorsal colour and head shape is underpinned by allelic differences. We also note that if underlying allelic differences are present then the replication of the beach-inland patterns across the four areas clearly favour selection rather than drift. Colour variation in three North American lizard species across extreme white gypsum, dark lava flows and more typical dark brown background colours does not seem to be explained by phenotypic plasticity^32^, although the inclusion of both gypsum and lava habitats in that study provided more divergent substrate colours than those described here (i.e., Madeiran grey shingle beaches versus vegetated inland sample areas). Other lizard studies have identified distinct alleles that appear to underpin geographic variation in dorsal luminance/melanism^23,33^. In other cases, while divergence in specific genes explained darker Utah lizards on dark volcanic lava flows, simulations also suggested that phenotypic plasticity might have facilitated differences in melanism prior to *de novo* mutations appearing^34^. The latter finding could be applicable to beach-living *Teira dugesii*, i.e., observed divergence represents only the phenotypic stage of this process. However, the considerable variance in dorsal luminance of beach-living populations seems to indicate incoming migration by ‘inland’ alleles (as supported by our simulations): a short term plastic response should more likely lead to dark coloration in all beach lizards. Specific mutations that influence melanin production and underpin variation in dorsal luminance have been identified in several other lizards^23,33,34^ giving weight to the hypothesis that allelic differences in relevant genes (such as *MC1R*) are responsible for the divergence in luminance here.

Genetic components of variation in head morphology are less well-established, although it has been reported that a substantial proportion (i.e., over 50%) of the variation in head morphology of the wall lizard *Podarcis muralis* is likely to be inherited^35^. Studies that identify potential genomic regions that might underpin these morphological characteristics are clearly needed.

In vertebrates, between-population divergence in colour mediated by divergent selection has been reported for several lizards from different habitats such as three species that have colonized white gypsum substrates at White Sands in New Mexico^24^, and also different species of *Peromyscus* mice in Florida and Nebraska^25,36^. *Tropidurus* lizard species in Roraima, Brazil, also show morphological divergence between populations from rock outcrops and savannah habitats^37^. However, while high gene flow is inferred in these cases^38^, the current findings are quite novel because the absolute geographic separation is substantially lower. Nonetheless, differences in dewlap colour have recently been described in island *Anolis* lizards in different habitats that are separated by only a few kilometres and likely to experience high levels of gene flow^39^, while microgeographic divergence has been described in several other taxonomic groups^40,41^. The very close proximity of the distinct beach and inland populations facilitates very high gene flow which should in turn dilute the effects of selection. Little is known about dispersal rates although ranges of introduced populations of another wall lizard (*P. muralis*) seem to extend by approximately 40–70 m per year^42^ which is high relative to the separation between our sites. A non-vertebrate example shows how very divergent selection can cause divergence in the face of high gene flow. The marine gastropod *Littorina saxatalis* differs between intertidal shoreline habitats that can be as close as ~10 m apart^40^, although migration rates must be much lower than those in *Teira dugesii*.

There are occasional reports of other lizards that inhabit the shoreline, includingisland wall lizards^43^, other island squamates such as skinks^44^, *Uta*^45^, *Microlophus*^46^ and the well-known Galapagos marine iguana which is intertidal/subtidal^47^, but to our knowledge, morphologically divergent intertidal populations have not been described. Future studies will be useful in determining whether the same mutations underpin divergence at different localities or not. For example, it is feasible that some or all of the described divergence is due to changes in allele frequency at the same loci.

Irrespective of the genetic basis, there is some variation between mean estimates of initial timing of divergence which suggests that beach colonization may have occurred at different times across the four localities despite the degree of beach-inland morphological variation being similar. Several recent studies of morphological differences between rural and urban environments^48–50^ indicate that these estimates of divergence times are long relative to the short times under which morphological divergence becomes detectable.

At present we can only speculate on how divergent selection might operate in different habitats. Broadly, lower dorsal luminance (more melanic) could enhance crypsis on the darker beaches, as originally postulated by Davenport and Dellinger^26^, while a brown/green coloration might be a better match to inland habitats. During fieldwork, Kestrels (*Falco tinnunculus*) were seen nesting and hunting by the coast. Predation by this species is thought to be quite intense^51,52^ and could be a potential driver of selection on dorsal colour. The fact that both males and females are darker on the beach suggests that sexual selection is not principally involved in determining colour differences between habitats. There are several possible explanations of divergent selection on head morphology, but it would be speculative to consider these until further data have been collected.

Overall, this study shows that within-island divergence can originate from differences between habitats alone, without requiring interruption to gene flow. Island topographies, particularly elevations, can lead to extremely heterogeneous environments and this variation is often correlated with within-island variation in lizard morphology^53–55^. We show that environmental differences between beach and inland habitats may have a much greater impact on morphology than do other quite substantial environmental differences across inland sites^56^. We also more generally suggest that substantial within-island morphological divergence is most likely to arise when there is either (i) divergent selection that is strong enough to overcome gene flow and may originate following colonization of a novel environment (such as the shoreline) as shown here, or (ii) historical population fragmentation that has impeded gene flow, as shown by previous studies.

## Methods

### Study species

The native lizard *Teira dugesii* is endemic to the Madeira archipelago in the Atlantic and its high abundance has been well-documented^57^. It is found across most habitats in the island of Madeira (maximum elevation 1862 m a.s.l, surface area 742 km^2^) from sea-level to the highest peaks where it lives in rocky refuges. It is diurnal in habit and eats invertebrates and vegetable material^58^. Environment-correlated patterns of morphological variation are evident but appear quite weak relative to other oceanic island lizards^56^. Also, there is no evidence of strong within-island phylogeographical patterns^59^ unlike some other insular systems where distributions of divergent ancient lineages are concordant with morphological variation^60^, making interpretation more complex. This study builds on previous work that described a melanic population from a grey shingle beach in south-east Madeira, i.e., Caniço^26^. While there are a small number of reports of lizards that inhabit the seashore, e.g., ref. ^61^, the finding of *Teira dugesii* living in the intertidal zone was a fairly interesting observation. In addition, the described population displayed morphological characteristics that appeared to be adaptive, such as darker skin pigmentation^26^.

### Sampling

**Fig. 5:**
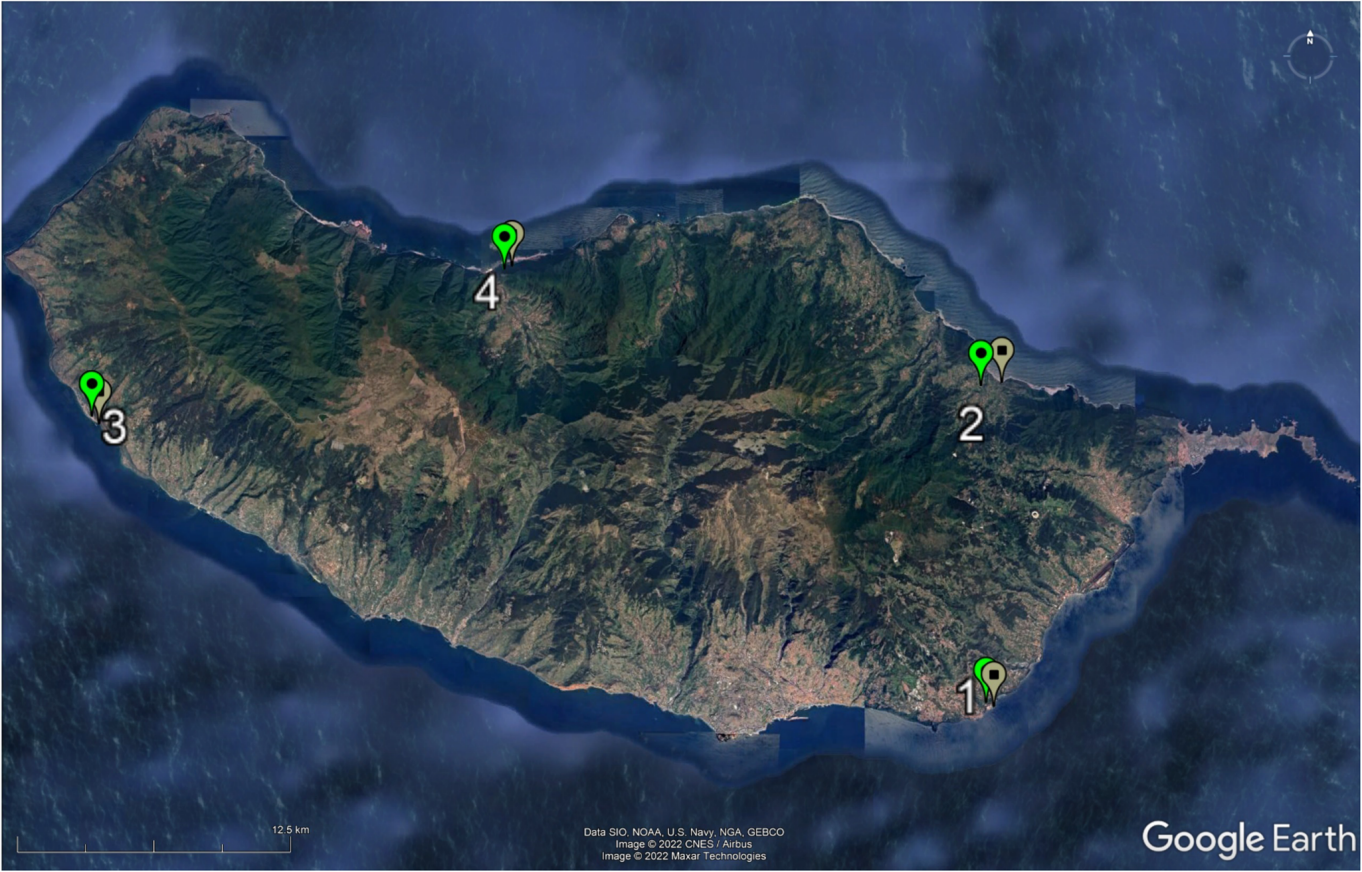
Sample sites on Madeira. Google Earth Pro v.7.3.4.8642 image showing the four sampling localities (1–4). At each locality, lizards were sampled from one beach (light green placeholder) and one inland (grey placeholder) site. Latitudes and longitudes are proved in the Supplementary Information.

Animal ethics: the study was approved by Liverpool John Moores University Animal Ethics committee on 05/06/19 and fieldwork was authorized by the regional government of Madeira (IFCN – DSGFB, capture permit 10/IFCN/2018 – FAU MAD). A matched pairs design was used with B and adjacent I habitats being identified at four localities in different parts of the island (labelled 1–4, see Fig. 5, Supplementary Table 1). The B sites were all similarly composed of mixtures of grey shingle/cobbles/boulders (see Supplementary Fig. 1). Traps were placed either within the shingle/cobbles and/or against the sides of boulders. Inland sites were less than 1 km away (see below) and were disused, overgrown coastal agricultural sites, where *T. dugesii* reaches very high densities^62^. All I sites contained loose stone walls, which provide refuges for the lizards. Traps were set along the sides of walls. Locality 1 (Caniço) was selected because it corresponded to the area originally described by Davenport and Dellinger^26^. Localities 2 (Porto da Cruz), 3 (Paul do Mar), and 4 (São Vicente) contained similar B and I habitats but were all quite distant (range: 13–39 km) from locality 1. Distances between B and I habitats within localities ranged from approximately 0.2 km between 4-I and 4-B, to 0.8 km between 2-I and 2-B.

Lizards were trapped at each locality/habitat (216 males, 118 females; adults were selected as these could be reliably sexed in the field) using upright plastic containers baited with fresh tomato. Sample sizes were similar for each of the eight sites (range 35–46 individuals; see Supplementary Table 1). All individuals were photographed (described below) with tail-tips also being removed from 93 individuals (9–14 per site) and stored in DNA/RNA Shield (Zymo Research). Sampling was authorized by the Regional Madeiran Government (Fieldwork/capture licence 10/IFCN/2018 - FAU MAD, issued on 04/12/18).

### Lizard dorsal luminance

The dorsa of all lizards were photographed using a Nikon D3300 camera with a zoom lens set at a focal length of 140 mm. Photographs were taken against the same background and included a standard 24-patch colour reference target (X-Rite ColorChecker Passport Photo 2) with scale bar. From each photograph, overall luminances were determined for the six dorsal/head areas (see below) from the three RGB channels using the multispectral imaging plug-in Micatoolbox v. 1.22^63^ within the programme ImageJ 1.52v^64^. Images were first normalized using known grey reflectance values for two of the X-Rite ColorChecker grey standard targets (10.17% and 59.41%). The six body characters on each lizard (four characters from the dorsal and lateral parts of the upper thorax and two characters from the head: Supplementary Fig. 2) were selected to represent variation in darkness of the head and upper part of the body. Character positions were located on all specimens and then the Micatoolbox plug-in was run on all normalized images, allowing extraction of mean pixel luminances.

RGB luminance was log_10_-transformed and significance of variation between localities and habitats was tested using a two-way MANOVA (IBM SPSS Statistics v. 26), following analyses of normality and equality of covariance matrices. Pillai’s trace test statistic was used for both males and females because the residuals of one of the six characters appeared to deviate from normality for both sexes and there was evidence of inequality of covariance matrices. General divergence was also explored using Discriminant Function Analysis (DFA), with individuals grouped according to the eight locality/habitat sites (sexes were analyzed separately due to sexual dimorphism). Overexposed photographs (corresponding to two males) were not used, so 214 males and 118 females were analyzed (see Supplementary Table 1 for details).

### Lizard morphology

Head measurements were taken from 2D images obtained in the field using a tripod-mounted Nikon D3300 SLR camera with a 60 mm Nikkor micro lens. Dorsal views of heads were photographed from a height of 30 cm with each photograph containing a scale bar. Previous laboratory testing showed that this protocol produced <5% measurement error compared with linear measurements taken using callipers. Five of the sampled individuals were not analyzed because photographs were subsequently deemed as insufficient quality so final sample sizes were 213 males and 116 females (see Supplementary Table 1 for full details).

Variation in male and female head morphology was captured using thirty-five landmarks with the programme tpsDig^65^ (Supplementary Fig. 3). All landmarks were recorded between the intersection of scale patterns, i.e., they were type 1 landmarks^66^.

Unless stated otherwise, morphometric analyses of size and shape variables were performed using the programme MorphoJ^67^. Males and females were analyzed separately. The 2D landmark coordinates were used to quantify head size as the centroid size (CS), which is defined as the square root of the squared distances between each landmark and the barycentre of landmark configuration^66^. Generalized Procrustes Analysis was applied following the established geometric morphometric protocol^68^ to standardize 2D coordinates, after translation, rotation and scaling to the unit centroid size. This generated two covariance matrices, corresponding to the symmetric and asymmetric components of shape^69^. The latter was discarded from further analyses while the symmetric covariance matrix, which explained the larger percentage of the biological variation within our sample, was used for Principal Components Analyses (PCA: see below).

For head size, log_e_ transformed CS was tested for the main effects of habitat and locality and habitat-locality interaction using two-way factorial ANOVA with IBM SPSS Statistics v. 26. For shape, the PCAs were used to obtain Principal Component (PC) scores that were used for subsequent analyses of head morphology. A two-way MANOVA (IBM SPSS Statistics v. 26) tested for locality and habitat effects on the PCs that represented 95% of the total variation, i.e., we disregarded the PCs that represented least variation. MANOVA assumptions were examined, as for the dorsal luminance data. A DFA was applied to the shape data (as represented by Procrustes coordinates) grouped by the eight locality/habitat sample areas.

### Habitat variation

Nine or ten standard photographs were taken at each locality/habitat to provide a simple assessment of differences in substrate luminance and vegetation cover. Each photograph was taken adjacent to a trap at which lizards were captured. Photographs contained a standard grey balance target (X-Rite ColorChecker Passport Photo 2) and a square wire quadrat (0.25 m^2^). Variation in substrate colour was assessed by comparison of means of RGB channels across quadrats, with site and locality as factors, using two-way MANOVA. Percentage vegetation cover was also recorded and compared between sites and localities.

### Genomic data

General genomic divergence between sites was established using genotype-by-sequencing (GBS), carried out as follows by Hangzhou Lianchuan Biotechnology Co., Ltd. Total genomic DNA was extracted from the lizard tail tips. The DNA was incubated with the restriction enzymes *ApeK*I and *Pst*I (NEB, Ipswich, MA, USA) at 37 °C and digested DNA then recovered using magnet beads and the GBS library prepared using the NGS Fast DNA Library Prep Set (Illumina, SanDiego, CA, US). The library was purified and electrophoresed on a 2.5% agarose gel and DNA fragments of 350–450 bp were excised and diluted before paired-end sequencing on a NovaSeq 6000 platform (Illumina, SanDiego, CA, US). Quality filtration was carried out; adapters were removed using AdaptorRemoval v2 (Schubert et al., 2016), and reads with low quality eliminated using FastQC v0.10.1^70^.

SNPs were called from the reads that were aligned using a GBS SNP Calling Pipeline (GBS-SNP-CROP v.4.1^71^). A minimum phred score base call quality of 30 was specified. Due to the lack of a reference genome, a mock reference was created from the individual with the greatest number of reads^72^. Following production of the variant discovery matrix containing all SNPs, variants were filtered largely using the default options except for the following: (1) alternate allele strength parameter (-altStrength) = 0.95, (2) maximum average depth of an acceptable variant (-mxAvgDepth) = 30, (3) minimum average depth of an acceptable variant (-mnAvgDepth) = 3, 4) minimum acceptable proportion of genotyped individuals to retain a SNP position (–mncall) = 0.90. SNP positions that also showed major heterozygote excess were also removed using VCFtools v. 0.1.16^73^: these were defined as SNP positions that showed a significant deviation from Hardy–Weinberg equilibrium at the 1% significance level. We also subsampled the full dataset (ALLSNPs) to obtain a thinned dataset with only one SNP per tag (to remove interdependence of SNPs in close proximity) and with any SNPs that appeared to be under selection removed (see later)

Pairwise F_ST_’s between sites were obtained using the R package PopGenome^74,75^ on the thinned dataset. Structuring of genomic divergence was also explored using a Discriminant Analysis of Principal Components (DAPC, within the R package adegenet^75,76^), using the ALLSNP data. This involved computing a PCA first (homozygous SNPs coded 0 or 2 and heterozygous SNPs coded 1). PCs with the largest eigenvalues were then input into a DFA. The number of PCs that were retained was determined from comparisons of Root Mean Squared Error (RMSE) and Mean Successful Assignment Rate (MSAR) of individuals to groups following cross-validation (100 training sets sampled from the data).

Potentially divergent selection on all SNPs between B and I habitats was tested using a two-step process. We first detected outlying SNPs using the pcadapt package v. 4.3.3, within R^75,77^ on the ALLSNP data. Four groups were specified to capture the observed population genomic structure. In brief, this approach involves a PCA on the SNPs, regression of individual SNPs on the PCAs and then testing of whether each SNPs Mahalanobis *D*^2^ distance, derived from the regression coefficients, is significant or not (by comparison with a χ^2^ distribution). Outliers were defined as those with a minor allele frequency greater than 5% that had a Bonferroni-adjusted outlier *p*-value <0.1 (with the aim of including most outliers). In the second step, an association between these outlying SNPs and habitat variation (using allele frequencies across the eight groups) was tested using bayenv2^78^. This analysis used a covariance matrix estimated by Bayesian MCMC analysis of the thinned dataset (following 200000 MCMC iterations the final posterior covariance matrix was retained). The B/I environment at each locality/habitat sample was specified using a binary variable. Bayes factors were obtained for all SNPs. Due to the stochasticity of this MCMC analysis, ten independent runs (i.e., starting from random number seeds) were carried out with 1000000 MCMC steps in each.

Spatial structuring was also investigated using spatial PCA (sPCA), as implemented in the R package adespatial^75,79^ (multispati command), using site latitudes and longitudes. PCA scores were obtained from the thinned data were used as input. Spatial information was supplied through a connection network of distances between sites, which allowed B/I individuals from the same locality to be specified as neighbours and those from different localities to be specified as non-neighbours. Significant local structuring occurs when genetic differences between neighbours are greater than those between randomly-selected individuals (negative spatial autocorrelation) while global structuring occurs when genetic distances between non-neighbours are greater (positive spatial autocorrelation). Eigenvalue tests (9999 randomizations) were used to test for local and global structuring^80^.

The hypothesis that populations from B sites formed a separate lineage from the I sites was examined using Treemix^81^ which estimates a tree representing historical population splits from population allele frequency data derived from genome-wide SNPs. No outgroup was available so historical migrations could not be inferred (although it still allowed the main hypothesis to be assessed). The thinned dataset was used for this analysis and support for the observed splits was obtained using trees obtained from 1000 bootstrap replicates.

Joint folded site frequency spectra (SFS) were used to compare three B/I scenarios of divergence at each of the four localities using the programme fastsimcoal2 (v. fsc27^21^) which implements a maximum likelihood approach to predict the SFS under each scenario for subsequent comparison with the observed SFS. The scenarios that were modelled were: (i) divergence without subsequent gene flow (NOGFLOW), (ii) divergence followed by constant gene flow (ONEGFLOW), (iii) divergence followed by two different periods of gene flow (TWOGFLOW) to accommodate, say, higher gene flow after divergence but lower gene flow nearer to the present. All SFS were obtained from SNPs with no missing values for all individuals within the four B/I habitat pairs. To help decrease the number of SNPs that showed missing values, the three individuals with most missing SNPs were removed from each sampled habitat, except for site 4 where only two individuals were removed. Two sets of analyses were carried out for each B/I pair using: (i) within-locality datasets subsampled from the full dataset (these are referred to as ALLSNP datasets and used to obtain parameter estimates), (ii) within-locality datasets subsampled from the thinned dataset, excluding any outliers determined by the pcadapt analysis (referred to as INDSNP datasets and used for model comparisons). The greater number of SNPs in the ALLSNP datasets should provide better parameter estimation^21^, but non-independence of SNPs may affect the robustness of likelihood-based model comparisons. Another reason for using the ALLSNP datasets was that reasonable estimates of the number of monomorphic sites could be used, allowing a fixed mutation rate (here, 1 × 10^−8^ mutations/generation). The number of monomorphic sites was estimated by first calculating the reduction in the number of SNPs from the master matrix containing all potential SNPs to the final set of filtered SNPs. We then assumed that this reduction reflected the reduction from the total number of sites sequenced to the total number of sites used (i.e., those from which filtered SNPs were identified). Potential errors in inference arising from the estimation of monomorphic sites should be relatively small, because (i) the number of monomorphic sites hugely exceeded the number of SNPs and was similar for all matched pairs, and (ii) identification of the best gene flow model and relative comparison of parameter estimates between regions was more important than precise parameter estimation (interpretations do not depend on absolute values).

For both ALLSNP and INDSNP analyses, estimations of the parameters that produced the greatest likelihood under each scenario were achieved using 100 optimization cycles, with 2 × 10^5^ coalescent simulations used to approximate the expected SFS in each cycle. This was replicated 100 times, with the replicate with the smallest deviation from the maximum observed likelihood being selected.

For the INDSNP analysis the Akaike information criterion (AIC) was compared between models. We also assessed stochastic variation in likelihood estimation by rerunning the fastsimcoal2 analyses 100 times using the parameters obtained for our best model.

Confidence intervals for the ALLSNP parameter estimates were obtained using the parametric bootstrap. For each locality, 100 SFS were generated to reflect the observed amount of genomic data structured as 300 bp contigs, reflecting our illumina reads. The parameters of the best model ALLSNP (as determined from analysis of the actual dataset) for the locality analyzed were used to generate these bootstrap replicates. These SFS were individually analyzed using the observed SFS for each run starting from the values obtained from the best run with the real data, based on 1 × 10^5^ coalescent simulations, 50 optimization cycles, and 40 replicates.

### Statistics and reproducibility

Statistical analyses were carried out using the programmes described above. Sample sizes per site for the morphological and genomics analyses are given in Supplementary Table 1. Kolmogorov–Smirnov, Box’s test of equality of covariance matrices, and F-tests for heteroscedasticity were used in SPSS to examine the assumptions of the MANOVA and ANOVA tests, although results should not be heavily dependent on these data characteristics due to the use of robust test statistics and large sample sizes.

### Reporting summary

Further information on research design is available in the Nature Portfolio Reporting Summary linked to this article.

## Supplementary information

Supplementary Information

Reporting Summary

## Data Availability

All data presented in this manuscript have been archived with the Knowledge Network for Biocomplexity (https://knb.ecoinformatics.org/): 10.5063/F15B00W3. All derivations from these data are available from the corresponding or final author on reasonable request.
