# Supplementary material for: Life on a beach leads to phenotypic divergence despite gene flow for an island lizard
Source: Commun Biol. 2023 Feb 3;6:141. doi: 10.1038/s42003-023-04494-x (PMC9895042; doi:10.1038/s42003-023-04494-x)

Supplementary Information: Life on a beach leads to phenotypic divergence despite gene flow for an island lizard

Richard P. Brown[1], Yuanting Jin[2], Jordan Thomas[1], Carlo Meloro[1]

[1]School of Biological & Environmental Sciences, Liverpool John Moores University, Liverpool L3 3AF, UK.

[2]College of Life Sciences, China Jiliang University, Hangzhou, 310018, P. R. China.

Supplementary Table 1

Latitudes, longitudes of sample sites and the number of individuals used in the analyses of male and female luminance. The numbers of individuals used for the analyses of head morphology are given in parentheses after the corresponding values used for analyses of luminance, but only where these differed. The number of males and females used in the GBS analyses are also provided. Here and elsewhere, the beach sites at localities 1-4 are suffixed -B and inland sites are suffixed –I.

Site	Longitude	Latitude	Male luminance (& morphology)	Female luminance (& morphology)	Male GBS	Female GBS
**1-B**	16°49'27.26"W	32°38'45.40"N	20(19)	16	8	6
**1-I**	16°49'40.22"W	32°38'51.60"N	21	20(19)	7	2
**2-B**	16°49'14.12"W	32°46'2.87"N	34(33)	3	10	2
**2-I**	16°49'48.48"W	32°45'58.65"N	29	17	8	3
**3-B**	17°13'22.12"W	32°45'6.51"N	31	16	9	5
**3-I**	17°13'34.27"W	32°45'17.01"N	19	24(23)	8	4
**4-B**	17°2'24.23"W	32°48'40.22"N	35	4	8	3
**4-I**	17°2'31.15"W	32°48'38.33"N	25(26)	18	8	2

Supplementary Table 2

Standardized variable coefficients of lizard RGB luminance for colour characters (Char.) 1-6 showing the coefficients of discriminant functions DF1-DF6, for the male and the female discriminant function analyses.

	**Male discriminant functions**						**Female discriminant functions**					
Char.	DF1	DF2	DF3	DF4	DF5	DF6	DF1	DF2	DF3	DF4	DF5	DF6
1	.361	.026	-.988	-.320	-.604	-.113	.811	.730	-1.034	-.975	-.134	-.857
2	.327	-.518	-.091	-.574	1.010	-.519	-.026	-.250	.210	1.388	-.175	-.766
3	.223	.767	.768	-.262	.149	.089	-.368	-.068	1.705	.705	.104	.477
4	.434	-.275	.602	.174	-.589	.834	.632	-.818	-.183	-.826	-.194	.719
5	.121	.013	.401	.635	-.376	-.966	.401	.034	-.014	-.205	1.325	-.093
6	-.051	.541	-.426	.634	.514	.744	-.205	.835	-.329	.302	-.738	.795

Supplementary Table 3

Structure matrices from discriminant function analyses of the three elements (RGB) of substrate luminance for colour characters (Char.) 1-6 showing correlations with standardized canonical discriminant functions 1-6, for the male and female discriminant function analyses.

Colour channel	DF1	DF2	DF3
Red	-2.519	2.542	-2.334
Green	-.257	-2.096	4.375
Blue	3.049	.412	-1.571

Supplementary Table 4

Pairwise nucleotide F_{ST}'s from the thinned (i.e., one SNP per contig) SNP data between all site pairs.

Site	1-I	2-B	2-I	3-B	3-I	4-B	4-I
1-B	0.012135	0.013321	0.016530	0.013246	0.016486	0.012257	0.019308
1-I		0.014172	0.018459	0.014995	0.017409	0.015616	0.024009
2-B			0.008658	0.018411	0.014705	0.011442	0.013967
2-I				0.021341	0.009249	0.011275	0.010182
3-B					0.015335	0.016979	0.021983
3-I						0.015070	0.015477
4-B							0.009580

Supplementary Figure 1

Examples of inland (1-I), left and beach (4-B) sites

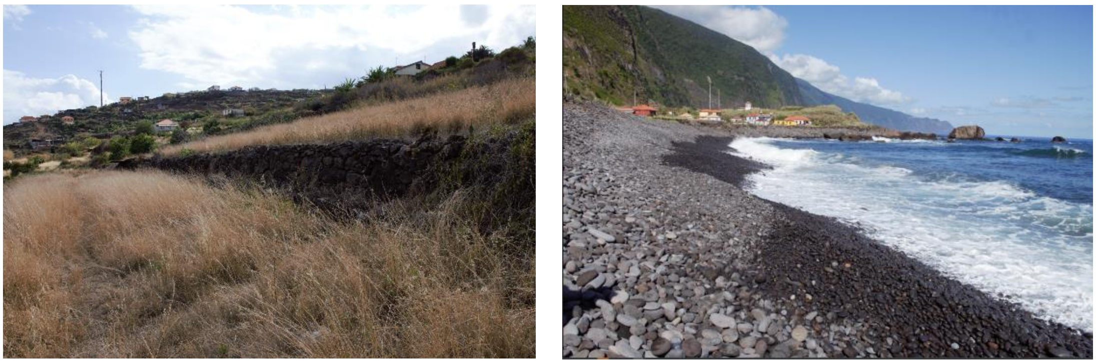

Supplementary Figure 2

Positions of the characters used to measure dorsal luminance on *Teira dugesii*. Characters 1 and 2 provide averages across 20x2 scales, character 2 on the central dark dorsum and character 1 on the lighter lateral stripe (the contrast between these regions is not clear on all specimens). These regions contain light and dark scales. Characters 3-6 are measured as the luminances of specific scales (see Methods). Character 3 is the luminance of a light scale within area 1, while character 2 is the same for a dark scale within area 2.

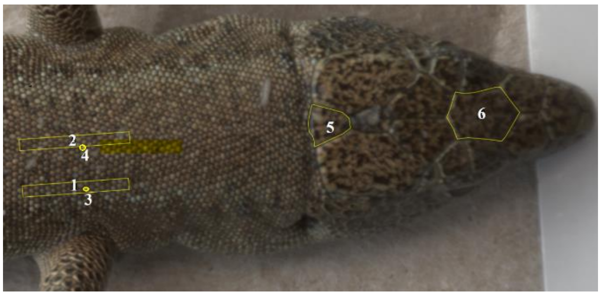

Supplementary Figure 3

Positions of the 35 dorsal landmarks placed on the heads

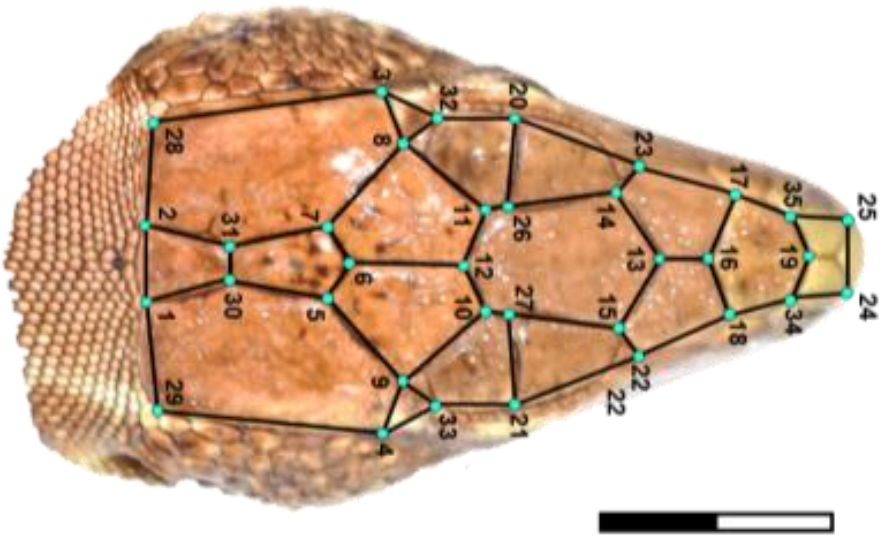

Supplementary Figure 4

Box-and-whisker plot of the percentage vegetation cover per quadrat recorded at each site. Site labels (x-axis) correspond to those in Figure 1. While vegetation was recorded at all inland sites (-I), no vegetation was recorded in any quadrat at the beach sites (-B). Asterisks indicate outliers.

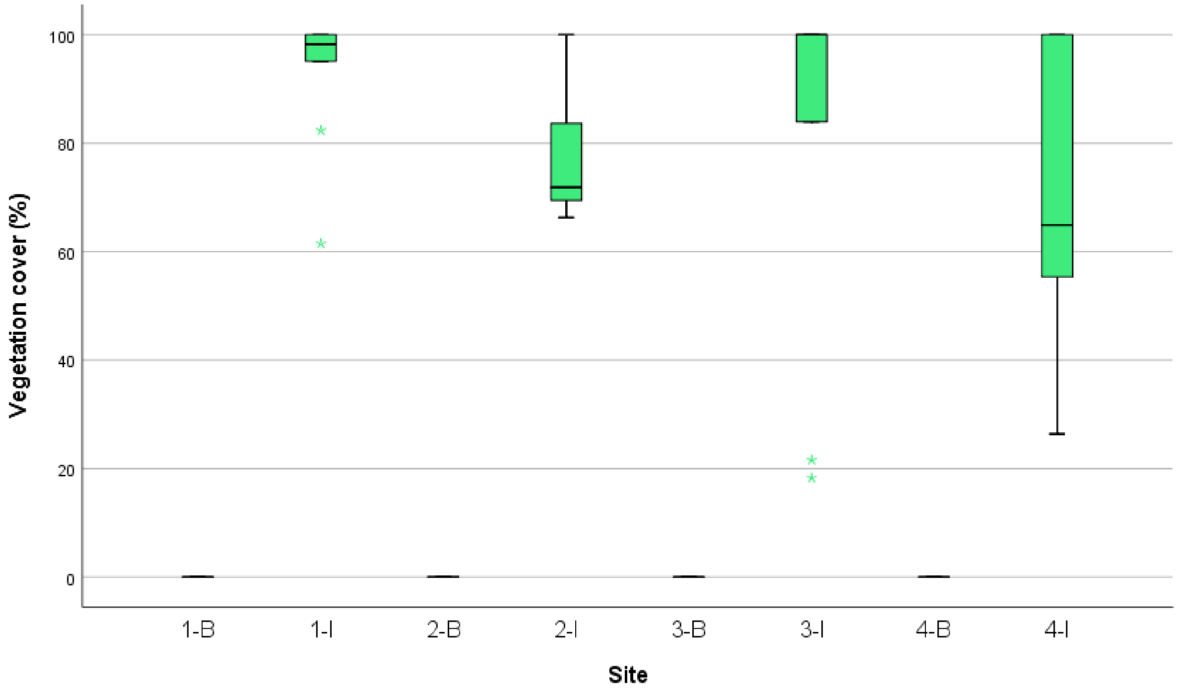

Supplementary Figure 5

A. Male Centroid size (log scale) at the four localities

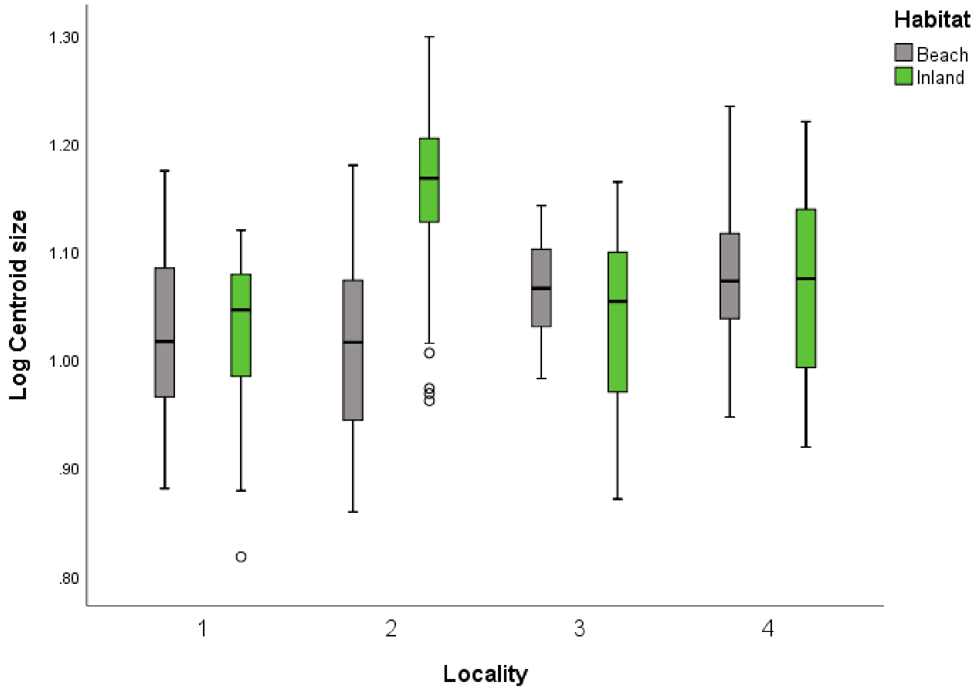

B. Female Centroid size (log scale) at the four localities

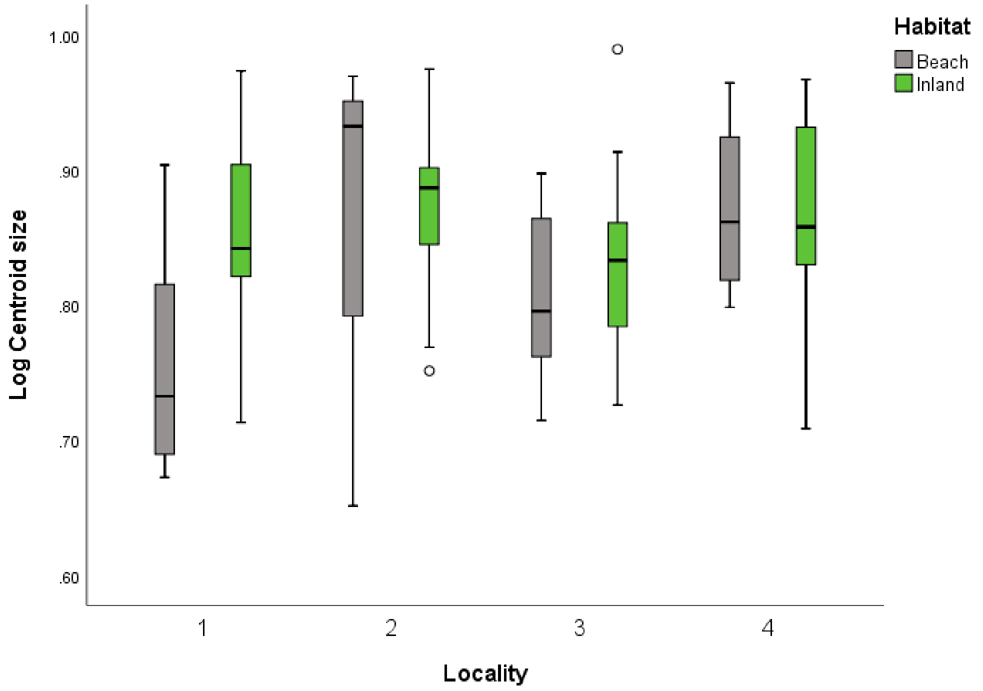

Supplementary Figure 6

Tree obtained using Treemix using GBS data. Branches are labelled with their bootstrap support values. Tips are labelled by site.

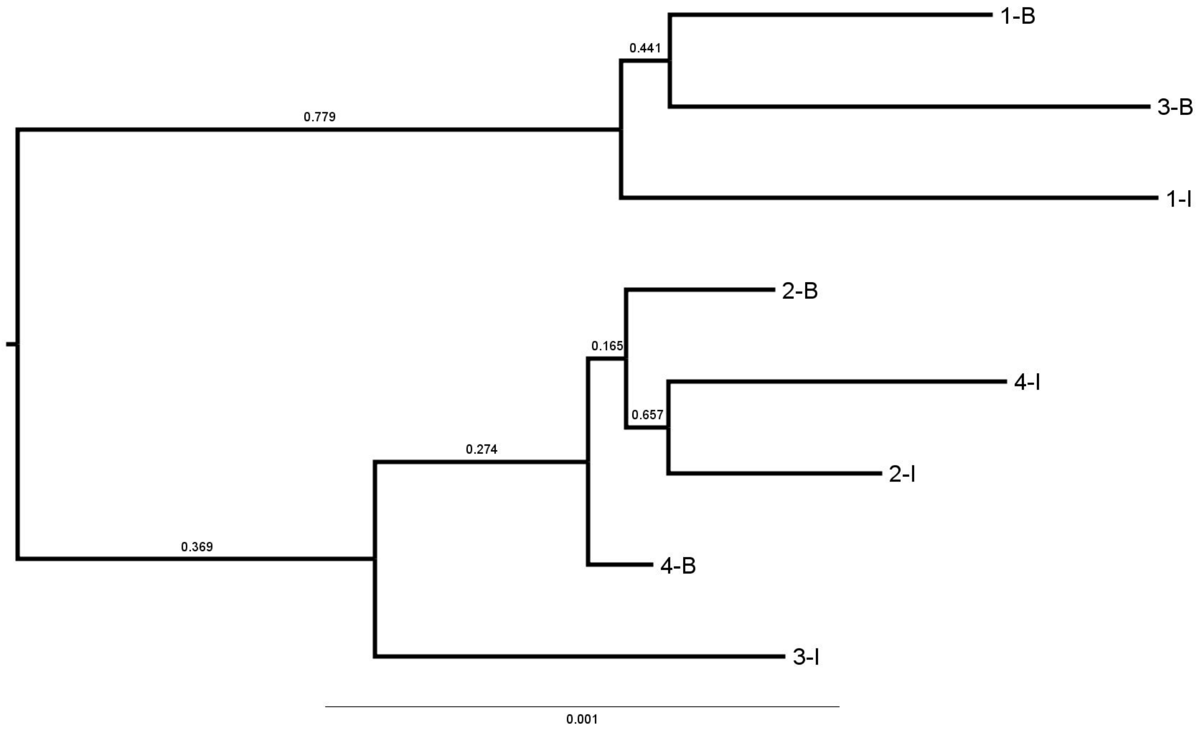

Supplement: Supplementary file 1 — Supplementary Information [file 42003_2023_4494_MOESM1_ESM.pdf]
